# Bilateral Crosslinking with Glutaraldehyde and 1-Ethyl-3-(3-Dimethylaminopropyl) Carbodiimide: An Optimization Strategy for the Application of Decellularized Human Amniotic Membrane in Tissue Engineering

**DOI:** 10.1155/2024/8525930

**Published:** 2024-04-24

**Authors:** Fatemeh Alibabaei-Omran, Ebrahim Zabihi, Alexander M. Seifalian, Nima Javanmehr, Ali Samadikuchaksaraei, Mazaher Gholipourmalekabadi, Mohammad Hossein Asghari, Hamid Reza Nouri, Roghayeh Pourbagher, Zinatossadat Bouzari, Seyedali Seyedmajidi

**Affiliations:** ^1^Cellular and Molecular Biology Research Center, Babol University of Medical Sciences, Babol, Iran; ^2^Department of Pharmacology and Toxicology, Babol University of Medical Sciences, Babol, Iran; ^3^Student Research Committee, Babol University of Medical Sciences, Babol, Iran; ^4^National Elite Foundation, Mazandaran Province Branch, Tehran, Mazandaran, Iran; ^5^Nanotechnology & Regenerative Medicine Commercialization Centre (NanoRegMed Ltd., Nano Loom Ltd., & Librium Health Ltd.), London Bioscience Innovation Centre, London, UK; ^6^Department of Medical Biotechnology, Faculty of Allied Medicine, Iran University of Medical Sciences, Tehran, Iran; ^7^Cellular and Molecular Research Center, Iran University of Medical Sciences, Tehran, Iran; ^8^Department of Gynaecology and Obstetrics, Babol University of Medical Sciences, Babol, Iran; ^9^Dental Materials Research Center, Babol University of Medical Sciences, Babol, Iran

## Abstract

**Introduction:**

The decellularized human amniotic membrane (dHAM) emerges as a viable 3D scaffold for organ repair and replacement using a tissue engineering strategy. Glutaraldehyde (GTA) and 1-ethyl-3-(3-dimethylaminopropyl) carbodiimide (EDC) can increase the biomechanical properties of dHAM. However, the crosslinking process is associated with biochemical changes and residual toxic materials, dampening the biocompatibility of the dHAM. From a histologic point of view, each side of the amniotic membrane is biologically different. While the dHAM basement membrane side is rich in growth factors, the stromal side of the dHAM contains more connective tissue matrix (e.g., collagen fibers) which supports its biomechanical properties. Biocompatibility and biomechanical properties are two important challenges in the field of materials science. In this study, for the first time, the stromal and basement membrane side are cross-linked with GTA and EDC, respectively, to optimize the biocompatibility of the treated dHAM while sparing the GTA-mediated biomechanical improvements.

**Methods:**

Crosslinking was carried out on dHAM in three groups: EDC, GTA and bilateral treatment with EDC&GTA. Mechanical resistance, degradability, and crosslinking measurements were performed on treated dHAM. The viability of mesenchymal stem cells (MSCs) on the scaffolds was evaluated by the MTT assay. The expression levels of surface markers and images of the MSCs were thoroughly studied.

**Results:**

The results obtained showed that bilateral treatment of dHAM with EDC and GTA increased mechanical resistance. Similarly, the evaluation of surface markers revealed that bilaterally treated dHAM sustains the stemness and viability of MSCs at a level equal to that achieved with EDC alone. The SEM images indicated that the MSCs maintained adhesion on EDC&GTA-cross-linked dHAM.

**Conclusion:**

The current study explores a pioneering treatment of dHAM, a material long recognized for its regenerative properties, in a novel context. This research delves into the utilization of dHAM cross-linked with EDC&GTA, demonstrating its optimized efficacy in tissue engineering. The enhanced crosslinking technique significantly alters the membrane's properties, amplifying its durability and therapeutic potential. In this novel bilateral treatment strategy (EDC and GTA), improving mechanical properties by GTA on the stromal surface and maintaining the biocompatibility of EDC on the side of the basement membrane of dHAM had been attained together. By investigating the handling and impact of this cross-linked membrane, this study unveils a new approach in leveraging a well-known material through an innovative process, revolutionizing its application in wound care.

## 1. Introduction

Tissue engineering (TE) is firmly committed to a promising horizon in regenerative medicine (RM) through biomaterial fabrication and tissue substitutes. A constellation of interconnected factors including the bioscaffold, stem cells (SCs), and growth factors are involved in the TE [1, 2]. The biophysical and chemical signals of the scaffold influence the characteristics and development of SC. Biomaterial science constantly seeks novel artificial and biological levers to create or enhance scaffolds in TE. Unlike conventional two-dimensional (2D) cultivation systems, these three-dimensional (3D) models offer a milieu similar to biological tissues with pronounced interplay between cells and cell-ECM, which is the cornerstone of the physiologic microenvironment, providing optimal cell growth and differentiation [3–5]. The biophysical architecture of artificial scaffolds is modifiable and, therefore, provides an optimum condition medium for the development of a particular tissue. Despite that, embedding synthetic scaffolds in TE and RM has been a challenging task because it does not exert structural competence similar to intricate organic materials [6]. Thus, it justifies the development of ECM-derived biologic scaffolds, which involves cell removal. Decellularization is related to the selective removal of natural cells and genetic compounds to isolate only the biophysical architecture and chemical materials. It paves the way for a new era of individual tissue grafting by cultivating patients' SCs on the ECM-derived scaffolds [7]. The latter has shown great improvement in the repair of a wide range of damaged tissues, such as the vasculature, cardiac valves [8], urogenital [9], hepatic [10], pulmonary [11], neural [12–14], skin [15, 16], cartilage [17], and ocular [11] severed tissues.

Given the limitations of synthetic materials, scientists started looking for more natural ways to help the tissue repair process. In this context, there has been a surge in the application of human amniotic membrane (HAM) in TERM. HAM has been widely acknowledged as a wound dressing for a century to treat chronic and acute wounds, such as burns [18], diabetic foot ulcers (DFUs) [19], and corneal defects [20]. Interestingly, the advantage of the collagen fiber network and ECM enriched with growth factors and cytokines makes AM useful as a bioscaffold for cell culture [21]. It is noteworthy to know that HAM surrounds the fetus and protects it against mechanical damage and infection. The HAM is a thin, colorless membrane without blood vessels, neural tissue, and myocytes. It forms the innermost layer of the extraembryonic membranes and its thickness varies from 20 to 500 µm [22]. This membrane consists of five main sublayers, namely, epithelial layer, basement membrane, compact layer, fibroblast layer, and spongy layer [23]. The epithelial layer consists of ectodermal-derived epithelial stem cells (ESCs), which are in contact with amniotic fluid. This layer is an abundant source of laminin, actin, vimentin, cytokeratin, desmoplakin, and *α*-actinin, which play an essential role in maintaining the structure, shape, and permeability of cells. The basement membrane is rich in collagens, laminin, and fibronectin, reflecting its implication in cell proliferation and differentiation. Collagen type II and IV in the compact layer improve the mechanical properties of HAM against enzymatic degradation by providing a firm connection between epithelial and interstitial collagens [24]. The fibroblast layer, the thickest layer of the HAM, contains mesenchymal stem cells (MSCs) of mesodermal origin. The spongy layer is the outermost layer of the amnion that is in contact with the chorion. Elastin, mucin, proteoglycan, and glycoprotein are present in this layer, making it loosely connected to the chorion and facilitating the separation of the HAM [25]. The human amniotic membrane is strongly resistant to mechanical and proteolytic factors due to having various types of collagen (such as type I, III, IV, V, and VI) [26]. The presence of hyaluronic acid, biologically vital proteins (i.e., fibronectin, laminin, and collagen), and proteoglycans in HAM that act as ligands for integrin receptors provide a suitable substrate for cell adhesion and growth [27].

MSCs and ESCs are multipotent stem cells that stimulate cell formation and differentiation by releasing cytokines and various growth factors in the extracellular matrix (ECM) [28]. Interestingly, ESCs are recognized to release antimicrobial agents, such as *β*-defensin peptides. Different types of low molecular weight elastases, SLPI, and elafin lend credit to the rigorous antimicrobial properties of HAM that reduce the risk of infection risk at the wound site [29]. The HAM dramatically reduces the risk of fibrosis by abating inflammation at the wound site. Specifically, the HAM stromal matrix deactivates the expression of proinflammatory cytokines such as IL-1*α* and IL-1*β* [30]. The HAM creates a biobarrier for tissues. Also, by adhesion to the wound surface, HAM covers the ends of the nerve fibers, ergo, reducing the pain in the wound area [31].

The deepening gap between organ donors and individual patients is compounded by ethical concerns, which justifies the search for viable alternative approaches, such as HAM. However, the immunogenicity and risk of rejection of the intact HAM are the bottleneck that impeding its wide application in TERM. Decellularization is a master step in blocking AM antigenicity while exempting its mechanical and biochemical characteristics, which contributes to decreased cytotoxicity and improved SC adherence. Decellularization enhances the biocompatibility of the HAM [32]. dHAM harnesses the architectural and biochemical cues of the native cell milieu to provide a flourishing microenvironment for cell growth to fabricate ECM and bioengineered tissues in cell culture and RM, respectively. There are various techniques to carry out the decellularization process, i.e., chemical, enzymatic, and physical, that show varying degrees of competence in terms of toxicity and protein perseverance, offering elastic modulus and tensile strength comparable to intact AM [33]. The reduction in the mechanical characteristics of dHAM is a challenge in TERM. To explain, after cell removal, collagenase released from cell debris interacts with ECM proteins, leading to vast degradation that impairs biophysical features [34]. For a more detailed explanation, collagenase enzyme activity increases at the wound site. This causes the amniotic membrane to degrade faster by destroying the collagen structure of the ECM. Despite the ideal characteristics of the decellularized amniotic membrane in tissue engineering and regenerative medicine, its low biomechanical properties and high rate of biological degradation, before the completion of the wound healing process, are among the main challenges that limit the use of AM. Further research and development are needed to improve the biomechanical properties and reduce the degradation rate of dHAM for its optimal use in clinical applications [35]. In this space, cross-linkers take center stage to strengthen dHAM bonds to protect against enzymatic degradation and improve biomechanical properties [36]. Among the various cross-linkers investigated, GTA and EDC are widely used to increase the stability of the bioscaffold architecture. GTA can profoundly enhance the resistance of the bioscaffold although potential toxicity ensues in high concentrations and exposure durations. Compared to EDC, a prominent disadvantage in GTA administration is its significant implication in biocompatibility because of the covalent bonds. Congruously, GTA exerts more promise in lower concentrations because increasing its concentration results in an excess compaction of collagenous fibers and many superficial bonds, which in turn, by forming an external shield, inhibits its further penetration into the matrix. Conversely, EDC is more biocompatible (nontoxic biomaterial and does not negatively impact cell attachment or growth of cells) [37] but compared to GTA, the cost of weaker crosslinking density and mechanical traits [38, 39]. The integration of crosslinking methods, such as EDC&GTA treatment, addresses many of these drawbacks by enhancing the membrane's structural stability, prolonging its durability and providing a more standardized platform for tissue engineering applications. Although EDC as a cross linker does not change the biocompatibility properties, it is not effective enough to improve the mechanical properties [40]. On the other page, GTA enhances the mechanical properties with strong crosslinking with the cost of decline in biocompatibility. This study uses bilateral crosslinking with EDC and GTA to assess and optimize its challenges. The basement membrane is treated with EDC to maintain biocompatibility and the stromal side is treated with GTA to form stronger transverse bonds. In particular, the stromal side of dHAM, affected by GTA, is rich in type III, I, and II collagens, which play an important role in providing structural support and promoting tissue regeneration [41].

The conventional use of this membrane has undergone a transformation as a result of limitations. As mentioned above, dHAM has low mechanical properties and biodegradation, making it easily ruptured during use on wounds and handling during surgical procedures [23]. Furthermore, the inherent properties of intact AM, such as thickness, transparency, and tensile strength, can vary significantly among donors [42]. This variability poses challenges in standardizing its use for consistent and predictable outcomes in tissue engineering.

The dHAM exerts different histoarchitectural and biological behaviors on its basement membrane side compared to the stromal side. Considering that the side of the basement membrane of the dHAM provides better support for SC growth [23], in this study, for the first time, we investigate the use of EDC treatment on the basement membrane and GTA treatment on the stromal sides of dHAM, evaluating their impact on the biomechanical and cell support properties of the dHAM.

## 2. Materials and Methods

### 2.1. Ethical Considerations

Fetal membranes were collected after elective cesarean section at Rouhani Hospital, Babol University of Medical Sciences (MUBabol), Babol, Iran. Informal consent was obtained from donors' mothers. All processes were completed in accordance with the MUBabol Ethics Committee and the Human Tissue Guideline (IR.MUBABOL.HRI.REC.1400.163). All donors were screened for health conditions and communicable infectious diseases that could impact donor suitability. Stem cell experiments were performed in accordance with the ethics license of the Iran National Committee for Ethics in Biomedical Research (https://www.ethics.research.ac.ir).

### 2.2. Preparation of the Human Amniotic Membrane

All steps of HAM preparation were performed under aseptic conditions. After the amnion was separated from the chorion, the remaining blood clots were removed. HAM was washed several times with phosphate-buffered saline (PBS) containing penicillin-streptomycin (Pen-Strep) and stored in 1 : 1 DMEM: glycerol at −20°C.

### 2.3. Decellularization of HAM and H&E Staining

The decellularization of HAM was performed according to the well-established protocol [43]. In brief, HAM was incubated with 0.2% EDTA for 30 minutes and then treated with 0.5 M sodium hydroxide (NaOH) for 20 seconds. Finally, the HAM was immersed in 5% ammonium chloride (NH_4_Cl) and the remaining cells were removed with a scraper. To validate the decellularization process, we performed H&E staining on paraffin-embedded samples.

### 2.4. Preparation of Chemically Cross-Linked HAM

In the cross-linked group of EDC, dHAM was immersed in 30 ml of PBS containing EDC (Alfa Aesar, U.K.) at 25°C for 6 hours. The cross-linked concentration was balanced at 0.05 mmol EDC/mg AM. To form crosslinking in the dHAM-GTA group, dHAM was treated with 0.1% GTA (Sigma-Aldrich, U.K.) for 6 hours at 25°C. A novel two-compartment chamber design was developed to perform the bilateral treatment of dHAM with GTA and EDC. The AM was placed between the two compartments, in which the stromal side was exposed to GTA 0.1%, and the side of the basement membrane was exposed to 0.05 mmol EDC/mg AM for 6 hours at 25°C. It is also worth mentioning that this method underwent thorough checks multiple times until a reproducible laboratory setup was achieved.

### 2.5. Inhibition of GTA Cytotoxicity

To diminish the toxic effects of residual GTA, we first thoroughly washed the cross-linked membrane with PBS to remove unreacted GTA from dHAM. Second, a glycine solution was applied to minimize the cytotoxicity of GTA in dHAM. Unreacted residual aldehyde groups of GTA were blocked by embedding dHAM in 100 mM glycine (Merck) aqueous solution at 25°C for 1 hour after crosslinking [44].

### 2.6. Measurement of the Crosslinking Degree

The degree of crosslinking was evaluated using the ninhydrin assay. This method indirectly estimates the degree of crosslinking by determining the level of free amine groups in different samples. First, equal weights (1 mg) of each sample were heated with a 2 mL solution of ninhydrin (Sigma-Aldrich) solution (0.02 mg/mL) in a water bath at 40°C for 20 minutes. After being cooled to room temperature, the samples were diluted in 95% ethanol. The optical density (OD) of the solution was measured at 570 nm using a UV spectrophotometer. The glycine solution was used as a standard. The crosslinking index (%) of AM was determined by calculating as crosslinking index (%) = ((Cb − Ca)/Cb) × 100. The average of five independent measurements was reported as the final result. The level of free amine groups of each sample prior to and after crosslinking correlates with the OD (optical density) of the sample [45].

### 2.7. In Vitro Degradability

To measure the amount of biodegradation, first, 1 × 1 cm^2^ pieces of AM were dried and weighed (Wb). Each sample was immersed in degradation solution that included 1 ml of PBS containing 12 *μ*g of collagenase (Sigma-Aldrich, type I, clostridium histolytic). The samples were incubated in a shaker incubator with 50 rpm at 37°C and for four weeks. The degradation solution was replaced every week with the same concentration of collagenase. After four weeks, all degraded samples were taken and weighed (Wa) after drying. Lastly, the percentage of the remaining weight (Wa/Wb × 100) was calculated for each group [44].

### 2.8. Mechanical Test

The tensile test is one of the most common methods for evaluating mechanical strength. The biomechanical properties of the five groups were assessed using the universal testing machine (UTM). The samples were placed in a specifically revised testing device and strained with a 10 mm/min cross speed until rupture. The load cell (5 kg) recorded the maximum force before each case ruptured. The samples were preserved with PBS throughout the biomechanical test [46].

### 2.9. Isolation and Culture of BMSCs

In this investigation, BMSCs were used to assess the biocompatibility of AM. The extraction and culture of BMSCs were performed according to the standard protocol [47]. In brief, Wistar rats (less than one month old) were anesthetized and their femur and tibia bones were isolated. Then, both sides of the bones were cut and the bone marrow content was flashed out using complete culture medium. Finally, the BMSCs were transferred to the 50 ml flask and upon the third passage; the cells were used for in vitro examination.

### 2.10. Cell Culture on HAM Scaffolds

Scaffolds (4 × 4 cm^2^) were spread in a 24-well plate and sterilized by UV irradiation under sterile conditions for 30 minutes. Then, 2 × 10^4^ cell/cm^2^ were seeded on each scaffolds (*n* = 3). The implanted cells were incubated at 37°C, 5% CO_2_, and 95% humidity, and the culture medium was changed every 24 hours.

### 2.11. The MTT Assay

The 3-(4,5-dimethylthiazol-2-yl)-2,5-diphenyl tetrazolium bromide (MTT) assay was performed as previously described (43). The effects of chemically cross-linked AM on the viability of BMSCs were evaluated on days 1, 3, and 7. After removing the culture medium, cells were washed with PBS and incubated with a 5 mg/ml MTT solution for 2 hours. Cells were treated with DMSO for 20 minutes to dissolve the formazan produced. The OD of the formazan solution was measured using an ELISA plate reader at 570 nm, and compared to the control group, which included isolated cells without scaffolds.

### 2.12. Cell/Scaffold Morphology

The interaction of MSC cells with intact HAM, dHAM, and cross-linked dHAM (with EDC, GTA, and bilateral EDC&GTA) bioscaffolds was examined through electron microscopy. The morphology of the MSCs spread on different scaffolds was investigated after 72 h of incubation. All samples were washed with PBS, treated in a 2.5% GTA solution at 4°C for 12 h, dehydrated with increasing concentration of ethanol solution and placed in acetone (Merck) [48].

### 2.13. Flow Cytometry Analysis (Assessing Stemness)

The expression levels of CD markers in MSCs (CD45, CD44, and CD90) on the surface of the MSCs for each group were evaluated by flow cytometry. The MSCs were seeded in six-well plates at a density of 5 × 10^5^ cells/well for 48 h on all five types of scaffolds. The MSCs were seeded at the 5th passages (5 × 10^5^ cells) on all five types of scaffolds in a six-well culture plate and incubated for 48 hours. After the predetermined time points, the MSCs were harvested from the scaffold by trypsinization and suspended in PBS, separated from the scaffold by trypsinization, and suspended in PBS and then stained with phycoerythrin (PE)-conjugated antibodies against CD90, CD44, and CD45 (45). Finally, the presence of fluorescence was confirmed by FACSCalibur flow cytometer (BD Biosciences, USA) [49].

### 2.14. Statistical Analysis

All data were plotted and all statistical analyzes were performed using Graph Pad Prism 8. The data points on each graph represented individual samples, with the mean indicated by the central line and the error bars representing the standard deviation. Statistical differences of all tests in this study were determined using one-way ANOVA, Bonferroni post hoc test.

## 3. Results

### 3.1. Decellularization of HAM

**Figure 1:**
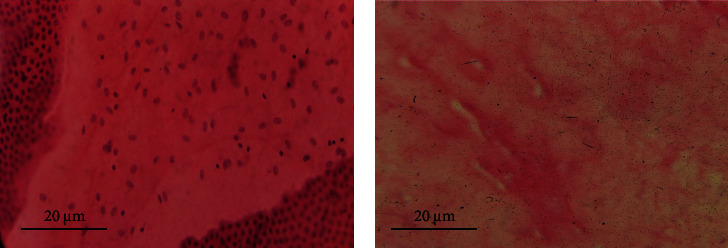
Hematoxylin and eosin (H&E) staining. (a) HAM and (b) decellularized HAM samples stained with H&E and photographed under a light microscope. The cells were successfully removed from the matrix after decellularization.

After HAM decellularization, H&E staining of HAM was used to confirm the success of the process. As shown in Figure 1, the decellularization process removed all cells from HAM (Figure 1).

### 3.2. Degree of Crosslinking

**Figure 2:**
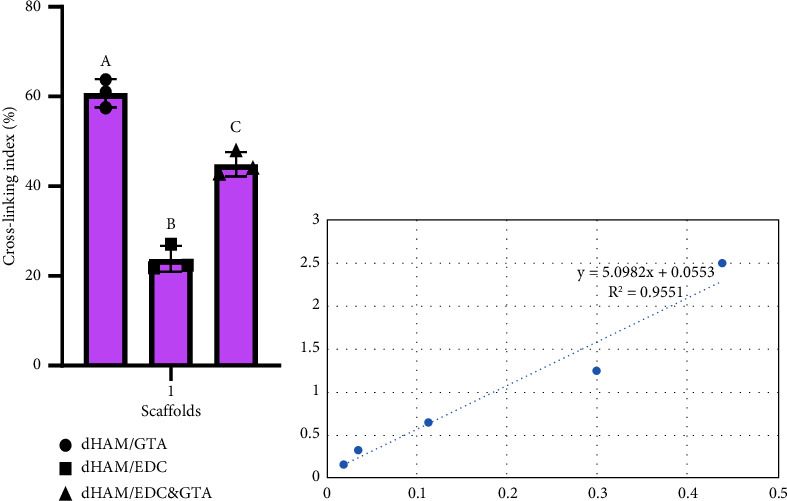
(a) Crosslinking index of various cross-linked dHAM. Different letters above the bars indicate a statistically significant differences at *p* < 0.0001. A one-way ANOVA was carried out to determine the statistical significance of the degree of crosslinking between dHAM/GTA, dHAM/EDC, and dHAM/EDC&GTA groups (*n* = 3), *F* (2, 6) = 120.7, *p* < 0.0001. (b) Glycine standard curve.

The ninhydrin assay was used to examine the amount of crosslinking of scaffolds created by each cross linker. The crosslinking index in dHAM/GTA, dHAM/EDC, and dHAM/EDC&GTA groups was found to be 60.8, 23.8, and 44.9%, respectively. There were significant differences between the dHAM/GTA group compared to dHAM/EDC&GTA (*p*=0.0014), the dHAM/EDC group compared to dHAM/EDC&GTA (*p*=0.003), and the dHAM/EDC group compared to dHAM/GTA (*p*=0.0003) in the degree of crosslinking (CLD) (Figure 2).

### 3.3. In Vitro Degradability

**Figure 3:**
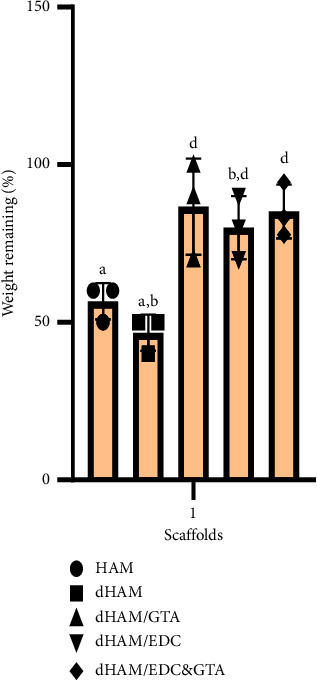
Percentage of the remaining weight of various scaffolds after incubation at 37°C for 4 weeks in a balanced salt solution containing collagenase. Different letters above the bars indicate statistically significant difference at *p* < 0.05 using the Bonferroni post hoc test. One-way ANOVA was carried out to determine statistical significance in the percentage of weight remaining among the HAM, dHAM, dHAM/GTA, dHAM/EDC, and dHAM/EDC&GTA groups (*n* = 3), *F* (4, 10) = 10.53, *p*=0.0013.

After four weeks of incubation of the samples in collagenase-digesting medium, the residual weight percentage for each sample was calculated (Figure 3). Based on the biodegradation results in the present study, dHAM, HAM, dHAM/GTA, dHAM/EDC&GTA, and dHAM/EDC exerted 46.6, 56.6, 86.6, 80, and 85.1% of their primary weight, respectively. The untreated dHAM groups exhibited a higher weight loss than the treated ones. According to the findings, the noncross-linked groups (dHAM and HAM) experienced similar levels of enzymatic digestion (*p*=0.719). Weight loss was reduced in the dHAM groups than in the dHAM/EDC groups (*p*=0.012), indicating that the crosslinking of EDC in dHAM helps prevent collagenase from destroying tissue. Compared to the dHAM group, the percentage of residual weight in the dHAM/EDC&GTA groups was statistically higher (*p*=0.031). On the other hand, the percentage of residual weight in the dHAM/GTA group was significantly higher than the dHAM group (*p*=0.003).

### 3.4. Mechanical Test

**Figure 4:**
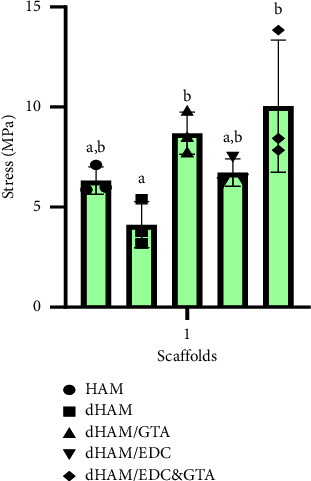
Ultimate tensile strength. Different letters above the bars indicate statistically significant differences at *p* < 0.05 using the Bonferroni post hoc test. One-way ANOVA was carried out to determine the statistical significance of mechanical resistance among the HAM, dHAM, dHAM/GTA, dHAM/EDC, and dHAM/EDC&GTA groups (*n* = 3), *F* (4, 10) = 5.434, *p*=0.0137.

Human amniotic membranes of 4 × 4 cm^2^ were produced from all groups. The test was carried out in triplicate and uniform tension (10 mm/min) was applied to each sample until rupture (the results are shown in Table 1). There is a statistically significant difference in the stress level (MPa) of the dHAM group compared to dHAM/EDC&GTA (*p*=0.01) and the dHAM group compared to dHAM/GTA (*p*=0.049). According to our findings for mechanical evaluation, bilateral treatment of dHAM with EDC&GTA (*p* < 0.05) enhances the biomechanical characteristics in a way comparable to the treatment of dHAM with GTA. On the other hand, in our experiment, the EDC could not significantly increase the mechanical resistance of dHAM (*p* > 0.05) (Figure 4).

### 3.5. Extraction and Passage of Mesenchymal Stem Cells from Rat Bone Marrow

**Figure 5:**
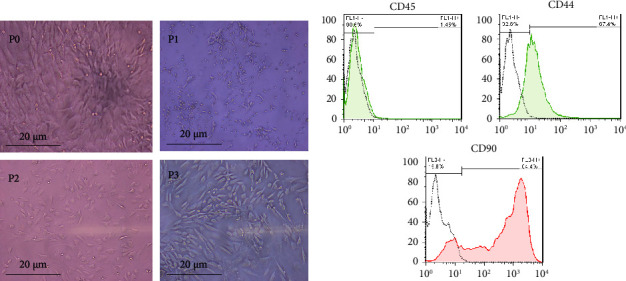
BMSC characterization. (a) The MSCs at the 3th subculture show spindle-shaped morphology. (b) Histogram representation of the fluorescence intensity flow cytometry results of CD44, CD45, and CD90 on the surface of the MSCs.

At passage zero, cell colonies of MSCs were visible. As cell passage increased, the percentage of purity of stem cells increased and they became spindle-shaped morphologically (Figure 5).

### 3.6. In Vitro Biocompatibility

The biocompatibility of AM treatments was performed at the following three levels: cytotoxicity assessment by the MTT test, the expression level of surface markers of MSCs by flow cytometry, and adhesion/morphology of MSCs by electron microscope.

#### 3.6.1. Cytotoxicity Assay (Viability)

**Figure 6:**
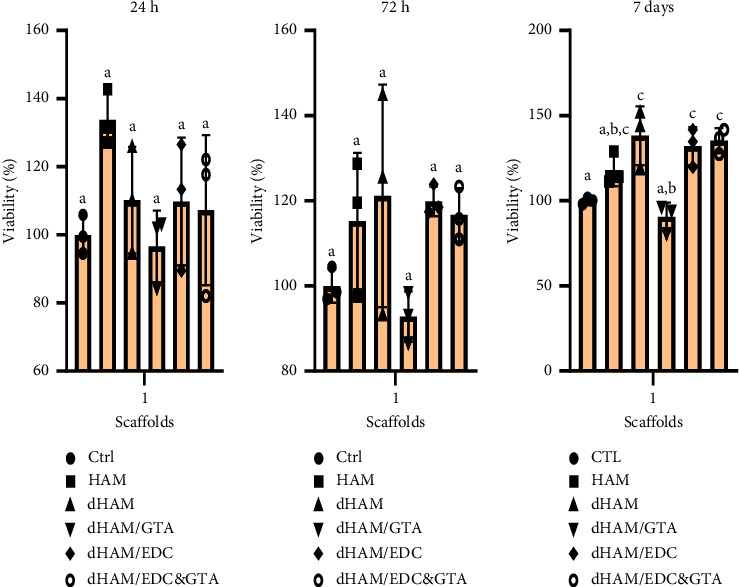
The cell viability of BMSCs was determined by MTT assay. 24 h, 72 h, and 7 days incubation of MSCs with various amniotic membrane scaffolds. Different letters above the bars indicate statistically significant differences at *p* < 0.05 using the Bonferroni post hoc test. One-way ANOVA was carried out to determine the statistical viability between the variation of the groups in 24 h of incubation (*F* (5, 12) = 2.383, *p*=0.0111), 72 h of incubation (*F* (5, 12) = 2.368, *p*=0.1206), and 7 days of incubation (*F* (5, 12) = 11.14, *p*=0.0004).

According to the viability assay, the scaffolds did not show toxic effects while they induced statistically significant (*p* < 0.05) cellular proliferation compared to the control (polystyrene). According to the MTT results (Figure 6), the viability of cells in dHAM/GTA after 24 and 72 hours did not differ significantly between the control group and the other groups. After one week of incubation, cell viability increased significantly on dHAM, dHAM/EDC&GTA, and dHAM/EDC scaffolds (138.2%, 135.3%, and 132.1%, respectively). Cell viability on dHAM/GTA after 24 and 72 hours did not differ significantly between the control group and the other groups 7 days after incubation. Cell viability on the dHAM/GTA scaffold was significantly lower than on the scaffolds dHAM (*p*=0.001), dHAM/EDC (*p*=0.003), and dHAM/EDC&GTA (*p*=0.002) after seven days, but there was no significant difference between cell viability on the dHAM/GTA and control groups.

#### 3.6.2. Cell/Scaffold Morphology

**Figure 7:**
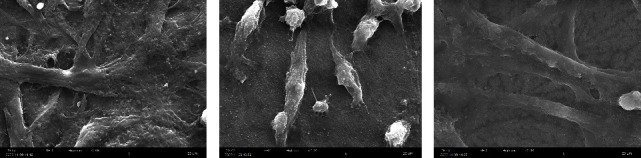
SEM images, morphology, and adhesion of MSCs on the scaffolds. (a) MSCs cultured on dHAM/EDC&GTA. (b) MSCs cultured on dHAM. (c) MSCs cultured in HAM.

SEM was used to examine the degree of adhesion, the morphology of cells on the scaffold, and the relationship between cells and scaffold. In the images obtained from SEM, MSCs on the dHAM treated with EDC&GTA were grown flourishingly, which indicates a successful culture of these cells on the surface of all AM derivatives (Figure 7). In addition, the images showed that the MSCs had preserved their morphology on the surface of the AM treated with EDC&GTA. Cells adhered to scaffolds (dHAM/EDC&GTA, dHAM, and HAM), but adhesion degrees were different. The cells on the dHAM scaffold had many extensions as cellular processes interwoven into the underlying membrane. On the other hand, cross-linkers masked the pores between the collagen fibers in the DHAM/EDC&GTA scaffold; therefore, the cells had less adhesion on this scaffold.

#### 3.6.3. Flow Cytometry Analysis (Stemness Assay)

**Figure 8:**
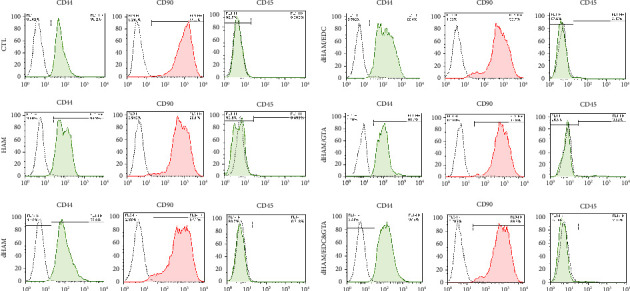
Immunophenotype of BMSCs cultured in polystyrene (as control), HAM, dHAM, dHAM/EDC, dHAM/GTA, and dHAM/EDC&GTA for 72 h, determined by flow cytometry analysis. Isotype control antibodies are represented by a gray line, and BMSCs are represented by a red line.

Flow cytometry analysis of bone marrow stem cells in the fifth passage showed CD90, CD44, and CD45. Surface markers revealed that they were MSC and nonhematopoietic (Figure 8).

The level of support provided by the scaffolds for the specific characteristics of the mesenchymal cells that were cultured on these scaffolds was evaluated using BMSC markers (CD90 and CD44) by flow cytometry. As expected, the surface antigen profiles matched the markers suggested by the recommendations of the International Society for Cell and Gene Therapy. Compared to the control group, the purity level of BMSCs in the scaffolds was not substantially different from that in the control group. The phenotypic examination of the BMSCs revealed that they lacked hematological markers (CD 45) (Figure 8).

## 4. Discussion

Decellularization is the process used to remove all cells and use tissue for clinical applications, such as a heart valve. Decellularization of the HAM removes epithelial cells on the fetal side of the membrane, which substantially reduces immunogenic reactions and improves its biocompatibility. Although complete removal of epithelial cells can be beneficial, it is more critical to maintain the composition of the ECM and the biomechanical characteristics of the HAM to promote biocompatibility. An industrial group in the USA used a decellularization HAM as wound dressing with positive outcome [50]. However, clinical application of these polymers has been slow, because of a lack of mechanical properties. Previously, we have developed a novel decellularization technique, which significantly reduced the DNA content of dHAM. Immunohistochemical studies on these membranes have shown that the most significant biological indicators of dHAM, such as type I, III, and IV collagens, were not alter by the process [43]. On the other hand, H&E staining was used in this investigation to provide a quality assessment of the decellularization process (Figure 1). It showed the small number of cellular remnants on the dHAM. The biocompatibility tests conducted on dHAM and HAM scaffolds revealed that this cell removal process did not significantly influence its biocompatibility (*p*=0.236), which supports its minimum effects on ECM compositions.

The optimal concentration of cross linkers has been a challenging issue; although some researchers suggested 0.05 mmol GTA per 1 mg dHAM [51], our preliminary results (not shown) demonstrated some stiffness and wrinkling in HAM when treated with 0.05 mmol of GTA. We found that the optimal cross linker concentration was 0.05 mmol EDC per 1 mg dHAM [51] and 0.1% GTA per 4 × 4 cm dHAM. This finding is consistent with the results obtained by Sporel et al., grafting the corneal surface with cross-linked dHAM. To optimize the crosslinking process, in this study, a low concentration of GTA is accompanied by EDC and is followed by glycine treatment to restore biocompatibility by quenching GTA-introduced aldehyde groups in the bioscaffold [52]. Our results also show that to reach the saturation level of crosslinking, 6 hours of treatment with EDC is sufficient (Figure 2). Meanwhile, the increase in GTA treatment time substantially boosts the CLD, at the cost of a steep decrease in biocompatibility (Figure 6). Interestingly, we found that dHAM/GTA had a higher CLD than dHAM/EDC. However, because in the dHAM/EDC&GTA group, the GTA reagent only made contact with the scaffold on one side (the stromal side), and its CLD was lower than that of dHAM/GTA alone and higher than that of dHAM/EDC alone. Although with different concentrations of GTA, an in vitro study conducted by Lai et al. demonstrated that during the first 6 hours of treatment, samples treated with GTA exert a significantly lower amount of crosslinking level compared to the EDC-treated group. Inefficient penetration of the cross linker into the protein matrix could be involved in this result because after 24 hours of treatment, GTA revealed higher levels of crosslinking and stability of HAM compared to the EDC-treated group [51]. The ninhydrin assay evaluates whether collagen fibers contain free amino acids that did not enter the reaction with cross-linkers. It has been observed that crosslinking reduces the amount of free amino acids in the HAM by creating bonds between the free amino acids of the collagen fibers. Thus, the higher the bond the cross-linkers create in the HAM, the less the reaction of ninhydrin with the free amino group would be.

Interestingly, independent lines of research indicated that crosslinking in the protein matrix of AM is an efficient strategy to hinder enzymatic degradation. This is partly due to the masking of certain sites of the dHAM bioscaffold by the bridges formed between the polypeptide chains by cross-linkers, thus making them unrecognizable by the active site of the enzyme.

**Figure 9:**
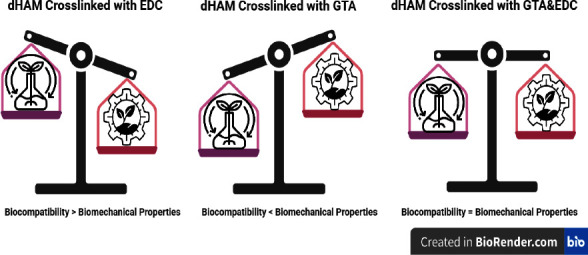
This schematic shows how bilateral (GTA&EDC) cross-linked dHAM could more efficiently maintain the balance between biocompatibility and biomechanical properties.

The degradability test shows that all three cross-linked groups (GTA, EDC, and EDC&GTA) have similar enzymatic resistance. In contrast, GTA and EDC&GTA create more mechanical resistance than the isolated EDC cross-linked group (Figure 9). On the basis of this, it is conceivable that GTA establishes resistance on both enzymatic and mechanical levels, while EDC crosslinking can only significantly increase enzymatic resistance. However, Lai et al. reported that after four weeks of exposure to collagenase enzymes, the percentages of the remaining mass in the samples treated with GTA and EDC showed statistically significant differences, which is in contrast to the results observed in the present investigation. Although EDC treatment (0.05 mmol/mg AM) resulted in a modest weight loss (17%) in the Lai's trial, weight loss in the group treated with GTA with 0.05 mmol/mg AM GTA was negligible. In addition to concentration, the duration of exposure to crosslinking agents is a vital factor concerning cytotoxicity [51].

The mechanical strength of the scaffold must be assessed since it experiences a substantial amount of stretch, tension, and handling stress, for example, in wound dressing applications. Many attempts have been made to improve the mechanical properties of the dHAM through crosslinking. We tested the mechanical strength using a tensiometer. The tensile strength of an intact HAM with epithelial cells reported 6.80 ± 0.22 MPa, which is consistent with the results of our study (Figure 4). Furthermore, the decellularization procedure in the present study sustained mechanical resistance postdecellularization similar to intact HAM levels (*p*=0.533). In addition, our mechanical evaluation results demonstrate that dHAM reinforced with EDC&GTA is as strong as dHAM reinforced with GTA (Figure 9). However, EDC alone did not considerably improve the mechanical strength of the dHAM (*p*=0.386).

The EDC does not integrate directly within the crosslinking bond; however, it activates the carboxylic acid groups of collagens to produce o-acylisourea groups, which, in turn, create cross-links through amide bonds between the free amine groups of collagen (zero-length crosslinking) [53]. However, EDC-cross-linked collagen is more susceptible to degradation by collagenase and its mechanical strength is not highly satisfactory. On the contrary, the GTA-cross-linked scaffolds show more enzymatic stability and mechanical strength compared to those treated with EDC. The GTA is a nonzero length cross linker that integrates directly within the bond, forming covalent connections (Schiff bases) between distinct amine groups, making the macromolecular network stiffer [54].

According to the outcomes of these tests, we believe that increasing the mechanical strength requires a strong crosslinking agent (GTA); however, even a mild to moderate bond, such as EDC-induced crosslinking, could substantially avert the enzymatic degradation.

The present study provides a comprehensive comparison of the effects of crosslinking dHAM with EDC and GTA, both isolated and in combination use for the first time to optimize the cross-linked dHAM composition for tissue engineering. The most challenging aspect of this study is improving the mechanical and enzymatic resistance while preserving the biocompatibility of dHAM. In this study, the viability, cell shape, and retention characteristics of MSCs were used as indicators of the biocompatibility of scaffolds made from amniotic membranes. The findings shed light on a novel strategy for abating the cytotoxic effect of the cross-linkers while simultaneously enhancing the mechanical properties of this widely used scaffold. Although HAM has an abundant source, a prominent obstacle in clinical transfer of HAM is its lack of substantial and ethical source of material. dHAM cross-linked with GTA and EDC provides promising enhancements in AM's biomechanical characteristics, such as increased physical resistance and decreased biodegradability. To further explain, maneuvering intact AM in laboratory settings is accompanied by several hurdles as it is viscous to instruments and hard to flip; however, cross-linked dHAM provides a more homogeneous appearance and is easier to handle, thus profoundly accelerating the efficiency of the procedures and reducing the tissue debris (unusable fragments).

Subtle changes in the chemical and physical properties of a scaffold can significantly affect cell proliferation and morphology. An overwhelming body of evidence has established that EDC exerts biocompatibility better than that of GTA because it does not change the molecular structure of the dHAM. Although the viability of MSCs on the dHAM/GTA scaffold was not substantially different from that of the control group (polystyrene plate) compared to that of dHAM, the viability rate of MSCs on the dHAM/GTA scaffold decreased substantially at the end of the first week. However, the ability to support the viability of MSC cells is not significantly different between dHAM/EDC and dHAM/EDC&GTA scaffolds (Figure 9). Cross-linked AM from EDC has been reported to have no cytotoxic potential and supports LEC proliferation (limbal epithelial cell) [39]. The dHAM/EDC&GTA scaffold is likely more successful at sustaining the viability of MSCs than dHAM/GTA. The absence of GTA crosslinking on the scaffold base membrane side, which cultivates MSCs, justifies its higher viability rate. Interestingly, EDC crosslinking in the basement membrane does not alter its molecular structure, thus maintaining the viability of MSCs. This is in line with the research carried out by Mirazul et al., as they examined the growth rate of the HCEC cell line in a double cross-linked collagen scaffold with GTA and EDC and reported higher mechanical and enzymatic resistance without toxic effects [55].

The expression of CD markers in the scaffolds was not substantially different from that of the control group, suggesting that all scaffold groups successfully supported the stemness qualities of MSCs. This is consistent with studies by Julien, who showed that cross-linking-induced microstructure modifications had no impact on the stemness of the hASCs [49]. In addition, SEM images demonstrate the substantial adherence and firm morphology of MSCs cultivated on the dHAM/EDC&GTA scaffold. Compared to MSCs cultured on the dHAM scaffold, which exert more perpendicular processes into the deeper layers, MSCs seeded on dHAM/EDC&GTA show a substantial amount of extended transverse processes due, in part, to the concealment of scaffold gaps by the cross-linkers, hindering in-depth extension.

## 5. Conclusion

Based on the results of this study, it can be concluded that the decellularization process effectively removes immunogenic epithelial cells while maintaining the composition and biomechanical properties of the ECM, leading to improved biocompatibility of the decellularized amniotic membrane (dHAM). In addition, the EDC&GTA cross-linked group has improved enzymatic mechanical resistance compared to the isolated EDC cross-linked group. The findings of this study suggest that the bilateral cross-linked decellularized amniotic membrane (by EDC and GTA) could be a suitable candidate for tissue engineering applications such as wound dressing and corneal tissue engineering. The conducting of a preclinical study under GMP (good manufacturing practices)/GLP (good laboratory practice) will be the next stage of this research before embarking on a clinical feasibility study for wound healing.

## Figures and Tables

**Table 1:** Tensile strength of decellularized human amniotic membranes after different sample treatments (*n* = 3) with EDC, GTA, and EDC&GTA.

Scaffold	Force (N)	Extension (mm)	Thickness (mm)	Stress (MPa) (mean ± SD)
HAM	7.679 ± 0.54	11.27 ± 6.08	0.026	6.319 ± 0.68
dHAM	4.210 ± 0.38	7.030 ± 5.18	0.024	4.119 ± 1.1
dHAM/EDC	3.760 ± 0.54	3.603 ± 2.19	0.028	6.714 ± 0.68
dHAM/GTA	6.130 ± 0.93	5.367 ± 5.18	0.024	8.686 ± 1
dHAM/EDC&GTA	9.510 ± 2.4	4.703 ± 1.65	0.023	10.068 ± 3

## Data Availability

The data used to support the findings of this study are available from the corresponding author upon reasonable request.

## Abbreviations

HAM: Human amniotic membrane
dHAM: Decellularization human amniotic membrane
EDC: 1-ethyl-3-(3-dimethylaminopropyl) carbodiimide
GTA: Glutaraldehyde
MSCs: Mesenchymal stem cells
BMSCs: Bone marrow stem cells
ECM: Extracellular matrix
SEM: Scanning electron microscope
CLD: Cross-linking degree
TERM: Tissue engineering regenerative medicine
TE: Tissue engineering
RM: Regenerative medicine
ESCs: Epithelial stem cells
SC: Stem cell
hASCs: Human amniotic stem cells.
